# Availability of Receptors for Advanced Glycation End-Products (RAGE) Influences Differential Transcriptome Expression in Lungs from Mice Exposed to Chronic Secondhand Smoke (SHS)

**DOI:** 10.3390/ijms25094940

**Published:** 2024-04-30

**Authors:** Katrina L. Curtis, Ashley Chang, Ryan Van Slooten, Christian Cooper, Madison N. Kirkham, Thomas Armond, Zack deBernardi, Brett E. Pickett, Juan A. Arroyo, Paul R. Reynolds

**Affiliations:** 1 Lung and Placenta Laboratory, Department of Cell Biology and Physiology, Brigham Young University, Provo, UT 84602, USA; 2 Department of Microbiology and Molecular Biology, Brigham Young University, Provo, UT 84602, USAbrett_pickett@byu.edu (B.E.P.)

**Keywords:** RAGE, RNA, tobacco, lung

## Abstract

The receptor for advanced glycation end-products (RAGE) has a central function in orchestrating inflammatory responses in multiple disease states including chronic obstructive pulmonary disease (COPD). RAGE is a transmembrane pattern recognition receptor with particular interest in lung disease due to its naturally abundant pulmonary expression. Our previous research demonstrated an inflammatory role for RAGE following acute exposure to secondhand smoke (SHS). However, chronic inflammatory mechanisms associated with RAGE remain ambiguous. In this study, we assessed transcriptional outcomes in mice exposed to chronic SHS in the context of RAGE expression. RAGE knockout (RKO) and wild-type (WT) mice were delivered nose-only SHS via an exposure system for six months and compared to control mice exposed to room air (RA). We specifically compared WT + RA, WT + SHS, RKO + RA, and RKO + SHS. Analysis of gene expression data from WT + RA vs. WT + SHS showed FEZ1, Slpi, and Msln as significant at the three-month time point; while RKO + SHS vs. WT + SHS identified cytochrome p450 1a1 and Slc26a4 as significant at multiple time points; and the RKO + SHS vs. WT + RA revealed Tmem151A as significant at the three-month time point as well as Gprc5a and Dynlt1b as significant at the three- and six-month time points. Notable gene clusters were functionally analyzed and discovered to be specific to cytoskeletal elements, inflammatory signaling, lipogenesis, and ciliogenesis. We found gene ontologies (GO) demonstrated significant biological pathways differentially impacted by the presence of RAGE. We also observed evidence that the PI3K-Akt and NF-κB signaling pathways were significantly enriched in DEGs across multiple comparisons. These data collectively identify several opportunities to further dissect RAGE signaling in the context of SHS exposure and foreshadow possible therapeutic modalities.

## 1. Introduction

Chronic Obstructive Pulmonary Disease (COPD) is a progressive and irreversible respiratory disorder characterized by the dual presence of chronic bronchitis and emphysematous destruction of lung parenchyma. COPD has been identified as the third leading cause of death globally, following only ischemic heart disease and stroke [1]. Despite significant advancements in our understanding of COPD, it remains incurable, with treatment strategies predominantly aimed at symptom relief. The therapeutic spectrum ranges from non-invasive interventions like bronchodilators, steroids, antibiotics, and oxygen therapy to more invasive procedures such as lung volume reduction surgery, lung transplants, and bullectomies, each tailored according to the disease’s severity [2]. Disease progression is primarily attributed to prolonged exposure to tobacco smoke or occupational hazards, whereas exacerbations are linked with long-term exposure to air pollutants or biomass particulates [3]. Chronic pulmonary inflammation, unregulated protease activity, and excessive mucus production are pivotal in driving the alveolar damage, impaired gas exchange, and airflow obstruction observed in COPD [4]. Consequently, patients experience a range of symptoms including persistent dyspnea, fatigue, sputum production, and coughing, which progressively intensify over time, potentially leading to mortality [5].

While primary smoking is a well-recognized risk factor for COPD, it is estimated that about one-quarter of COPD patients have never smoked [6,7,8,9]. Secondhand smoke (SHS) exposure emerges as a significant risk factor for COPD among non-smokers [3,10,11]. Despite a decline in SHS exposure in the United States, a considerable number of children and adults continue to be exposed to passive smoke in public spaces and homes [12]. Chronic SHS exposure is associated with an elevated risk of COPD mortality in adulthood, underscoring the need for continued public health efforts to reduce SHS exposure [13].

At the cellular level, the pathology of COPD involves numerous mechanisms and signaling pathways, with the receptor for advanced glycation end-products (RAGE) identified as a potential key player in the disease’s progression [14]. RAGE, a pattern recognition receptor (PRR) of the immunoglobulin superfamily, binds to a variety of ligands, including damage-associated molecular patterns (DAMPs) and advanced glycation end-products (AGEs), which are notably present in cigarette smoke [15,16,17,18]. RAGE activation initiates signaling cascades that lead to the nuclear translocation of NF-kB, thereby regulating genes related to immune and inflammatory responses [19,20]. These parallel pathways result in sustained inflammation, further exacerbated by RAGE’s expression in various cell types, implicating it in a range of diseases, including COPD. Previous research has highlighted RAGE’s role in acute pulmonary inflammatory responses to SHS, setting the stage for this study’s aim to explore the signaling pathways activated by RAGE during chronic pulmonary inflammation induced by SHS [21]. Understanding these pathways may unveil novel therapeutic targets for mitigating inflammatory exacerbations in COPD patients.

Studies utilizing RNAseq in COPD cohorts have identified novel genes and signaling pathways involved in inflammation, immune responses, and tissue remodeling [22]. Furthermore, RNAseq analyses have revealed significant alterations in gene expression associated with oxidative stress, protease–antiprotease imbalance, and apoptosis in the COPD lung [23]. Although transcriptomic studies that focus on COPD exist, none to date evaluate expression data in the context of controlled RAGE expression. We therefore sought to test the hypothesis that the availability of RAGE during sub-chronic or chronic exposure to SHS differentially regulates pathologic signaling events. Pursuing this will provide insights that will not only enhance our understanding of COPD pathogenesis but also pave the way for identifying potential biomarkers and therapeutic targets.

## 2. Results

### 2.1. Initial Findings

**Figure 1:**
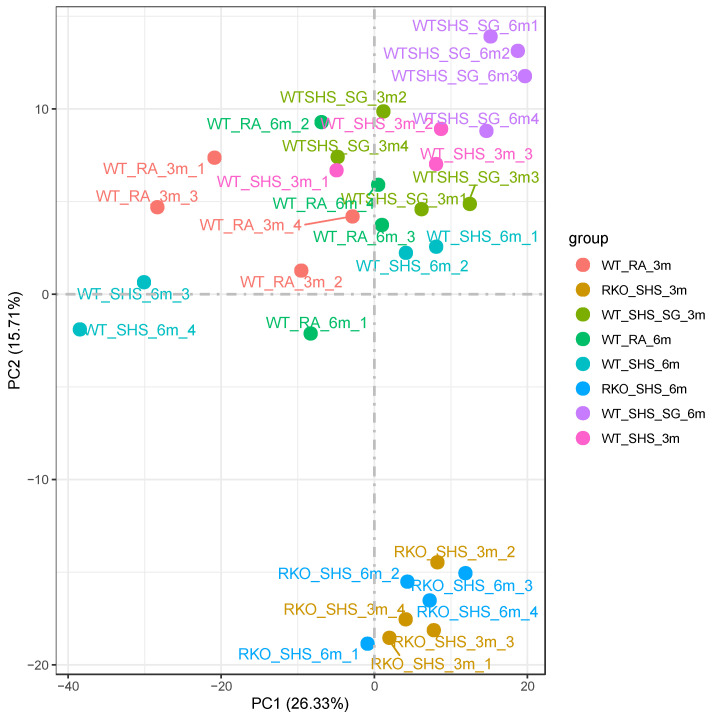
Principal Component Analysis Between All Samples. Visualization shows the quadruplicate samples tend to group primarily by WT or RKO, and secondarily by time point and exposure type. Relationships among the color-coded samples are shown in two dimensions.

**Figure 2:**
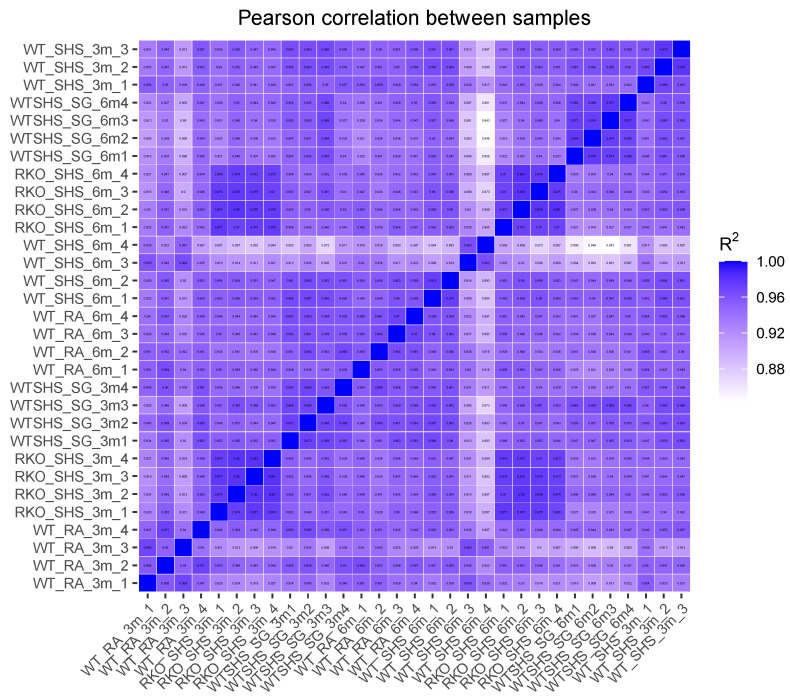
Correlation Analysis Between All Samples. Color-coded pairwise correlation values between all samples are shown. Samples having darker shading are more closely correlated. The diagonal, representing a self-to-self comparison, shows perfect correlation.

Given the relatively large number of genotypes represented by the samples (Table 1), we first wanted to better characterize the relationship between the various samples and comparisons using principal component analysis (PCA; Figure 1). As a recurring theme, we observed no differences between WT + RA and RKO + RA. Overall, we found that the samples with the same genotype, time point, and type of exposure tended to cluster together. To further characterize the associations between the various samples, we then performed a correlation analysis between all samples (Figure 2). In general, we observed that these correlations validate the PCA results.

In addition to identifying sets of differentially expressed genes (DEGs) that were either shared or unique between various comparisons (Supplementary Table S1), we generated a heatmap of the transcriptional results across all samples (Supplementary Figure S1). We also calculated the statistically significant pathways and Gene Ontology (GO) functional terms that were enriched in the genes for the same comparisons (Supplementary Table S2).

### 2.2. Comparison of WT + RA vs. WT + SHS Suggests Immune Component

We first examined the statistically significant DEG results between WT mice exposed to SHS vs. WT animals exposed to RA at the three-month and six-month time points. We found that the most statistically significant DEGs in these comparisons were Fasciculation and elongation protein zeta-1 (FEZ1; log2 FC at three months = 4.2, *p*-value = 1.01 × 10^−81^), Secretory leukocyte peptidase inhibitor (Slpi; log2 FC at three months = 3.97, *p*-value = 8.52 × 10^−33^), and mesothelin (Msln; log2 FC at three months = 4.67, *p*-value = 8.52 × 10^−33^), which were not found in the six-month time point. We also observed that Serine (or cysteine) peptidase inhibitor, clade A member 3C (SERPINA 3C; log2 FC at six months = −1.59, *p*-value = 7.83 × 10^−10^; not significant at three months) and endoplasmic reticulum protein 29 (Erp29; log2 FC at 6 months = −0.51, *p*-value = 2.29 × 10^−7^) were only significantly different in animals treated for six months.

When we analyzed the significant pathway results, we found pathways associated with the typical roles of RAGE, and others that were associated with cancer including “ECM–receptor interaction” and “Regulation of actin cytoskeleton”; as well as “Proteoglycans in cancer” and “Small cell lung cancer” at the three-month time point but were not significant at the six-month time point (Supplementary Table S2) Interestingly, there appeared to be a cluster of 11 significant pathways in the six-month time point that dealt with immune responses including “Viral protein interaction with cytokine and cytokine receptor”, “Asthma”, and “NF-kappa B signaling pathway”. We also found the “PI3K-Akt signaling pathway” to be significantly changed at the three-month time point (*p*-value = 0.0045).

### 2.3. Comparison of RKO + SHS vs. WT + SHS Suggests a Lipid Contribution to Damage

We then wanted to compare the RAGE KO animals exposed to SHS vs. WT animals also exposed to SHS at the three-month and six-month time points. This comparison would reveal biological responses to SHS in animals that expressed (WT) or did not express (KO) RAGE. As before, we first focused on the statistically significant DEGs.

The two most statistically significant DEGs at the three-month time point were advanced glycosylation end-product-specific receptor (Ager/RAGE; log2 FC at three months = −7.25, *p*-value > 0; log2 FC at six months = −6.17, *p*-value = 4.46 × 10^−22^) and cytochrome P450 1a1 (log2 FC at three months = −7.56, *p*-value = 1.54 × 10^−171^; not significant at six months). The most significant differentially expressed gene products at the six-month time point were solute carrier family 26 member 4 (Slc26a4; log2 FC at six months = 4.00, *p*-value = 1.20 × 10^−41^; log2 FC at three months = 2.66, *p*-value = 5.45 × 10^−11^). When we examined the signaling pathways for this set of comparisons, we found a subset that was both statistically significant and biologically relevant to the RAGE KO phenotype including “ECM–receptor interaction”, “AGE-RAGE signaling pathway in diabetic complications”, and “MAPK signaling pathway”. We also identified a set of pathways that dealt with lipids including “adipocytokine signaling pathway” at the three-month time point; as well as “Arachidonic acid metabolism”, “Fatty acid metabolism”, “Cortisol synthesis and secretion”, and “Ether lipid metabolism” at the six-month time point.

**Figure 3:**
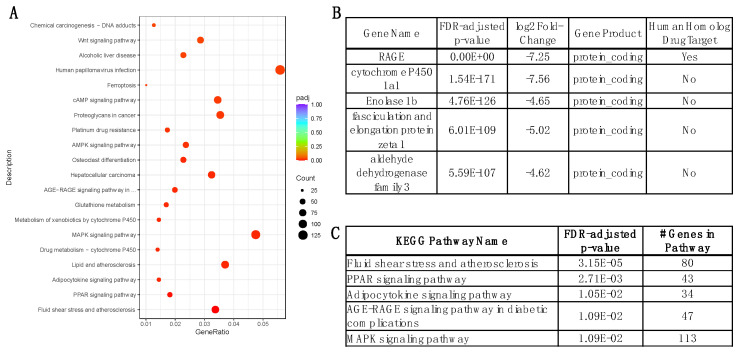
Most Significant Signaling Pathways and Differentially Expressed Genes in the Comparison of RKO + SHS vs. WT + SHS After Three Months: (**A**) Dot plot of significant signaling pathways between RAGE-knockout animals and wild-type animals after three months of exposure to secondhand smoke. Dot size and color represent number of genes in the pathway and the adjusted *p*-value, respectively. (**B**) Table showing the five most significant differentially expressed genes in a comparison between RAGE-knockout animals and wild-type animals after three months of exposure to secondhand smoke. (**C**) Table showing the top five most significant KEGG intracellular signaling pathways in a comparison between RAGE-knockout animals and wild-type animals after three months of exposure to secondhand smoke.

Comparing RKO + SHS vs. WT + SHS at the three-month time point has the highest potential to improve our understanding of the role and mechanism of RAGE. As such, we particularly focused on the most significant genes, GO functions, and signaling pathways (Figure 3).

### 2.4. Comparison of RKO + SHS vs. WT + RA

Lastly, we compared the RAGE KO animals that were exposed to SHS vs. WT animals exposed to RA at the three-month and six-month time points. Our analysis of the statistically significant DEGs at the three-month time point identified Ager/RAGE (log2 FC at three months = −5.90, *p*-value = 3.32 × 10^−97^; log2 FC at six months = −6.67, *p*-value = 0), and transmembrane protein 151A (Tmem151A; log2 FC at three months = 3.61, *p*-value = 3.31 × 10^−77^; not significant at six months). A similar analysis of the most significant DEGs at the six-month time point identified G protein-coupled receptor family C group 5 member a (Gprc5a; log2 FC at three months = 2.14, *p*-value = 1.68 × 10^−24^; log2 FC at six months = 2.47, *p*-value = 9.36 × 10^−110^) and dynein light chain Tctex-type 1B (Dynlt1b; log2 FC at three months = 2.56, *p*-value = 0.04; log2 FC at six months = 5.97, *p*-value = 1.46 × 10^−77^).

Our comparison of the significantly enriched pathways for these contrasts included a large number of pathways involved in inflammation and the immune response including “AGE-RAGE signaling pathway in diabetic complications”, “ECM–receptor interaction”, “Regulation of actin cytoskeleton”, as well as a subset that functions in lipid metabolism at the three-month time point. The comparison at the six-month time point included many of the same pathways revealed in the three-month time point assessment. In addition, the longer period of exposure coincided with the activation of more immune-related pathways including “Allograft rejection”, “Type I diabetes mellitus”, “Autoimmune thyroid disease”, “Rheumatoid arthritis”, and others. We also observed the PI3K-AKT signaling pathway to be significantly affected at both the three- and six-month time points (*p*-values = 0.014 and 0.033 respectively).

### 2.5. Multiple Comparisons Reveals Contribution of AKT and NF-kappa B Pathways

In addition to the above, we found additional evidence of an effect on AKT- and NF-kappa B-related pathways when we reviewed the other comparisons. Specifically, we observed that the “PI3K-Akt signaling pathway” was significantly affected in the 6 m WT_RA vs. 3 m WT_RA comparison (*p*-value = 0.001), and the “NF-kappa B signaling pathway” in the 6 m WT_RA vs. 3 m WT_RA comparison (*p*-value = 0.0184).

Our enrichment analysis also identified pathways that were statistically significant across multiple time points involving the RKO + SHS animals. For example, components of the “Human papillomavirus infection” as statistically significant in both time points of the RKO + SHS vs. WT + RA (*p*-values = 2.065 × 10^−6^ and 2.775 × 10^−6^) and the RKO + SHS vs. WT + SHS (*p*-values 0.0241 and 0.0149) comparisons. “Phagosome” was significant across both RKO + SHS vs. WT + RA time points and the later RKO + SHS vs. WT + SHS comparison, while both “ECM–receptor interaction”, “Cell adhesion molecules”, “Proteoglycans in cancer”, “Regulation of actin cytoskeleton”, and others were identified as significant across at least one time point in each of the three comparisons. We did not observe any statistically significant effects on apoptosis-related pathways in any of the comparisons.

## 3. Discussion

The goal of this study was to clarify possible intersecting roles for RAGE following sub-chronic (3 months) or chronic (6 months) exposure to SHS. To do so, we generated RNA-sequencing data and performed multiple computational comparative analyses that revealed a large number of differentially expressed genes and signaling pathways.

While this publication cannot address every DEG that we discovered in the analyses, our results did include several findings that have been shown previously. For example, the RAGE pathway was significantly affected in the KO animals, which confirms the phenotype of these animals and serves as an internal validation control in the relevant comparisons. In our comparison of WT animals exposed to either SHS or RA, finding SERPINA 3C as a differentially expressed gene agrees with past findings that SERPINA 3C is found in the extracellular space and is part of the response to various stressors including COPD [24,25]. The superfamily of Serpins is the largest, most broadly distributed group of protease inhibitors. They are extracellular molecules that regulate proteases involved in blood coagulation, inflammation, tissue remodeling, and immune responses [26]. We also found that the *Slc26a4* gene, which we showed to be significantly upregulated in both time points from the RKO + SHS vs. WT + SHS comparison, plays a key role in airway inflammation [27,28,29]. *Slc26a4* is a gene that codes for pendrin, a molecule that is often upregulated at the apical edge of airway epithelial cells involved in mucus overproduction [28]. The current work confirms a role for *Slc26a4*, most likely as a means of coordinating the expression of MUC5AC, a major product of mucus abundantly expressed during COPD progression, and further implicates a role for RAGE in its activity.

The discovery that lipid biology was differentially impacted by RAGE signaling supports a previously seen link between RAGE and fatty acid metabolism [30]. For instance, Wang et al. demonstrated that the loss of RAGE differentially impacted apoptosis and inflammation, which resulted in restored fatty acid oxidation [31]. Further, clarifying a link between RAGE signaling and the elaboration of adipocytokines further implicates RAGE in recently researched axes of metabolic syndrome, cancer, and obesity. Such connections include differential leptin and RAGE levels in patients with lung disease [32], chronic RAGE-mediated inflammation observed in cardiovascular metabolic disruption and neoplasia [33], and RAGE effects on adipocytokine levels in obese asthmatic mice [34]. These RNAseq data provide a compelling case for immediate research into the functional aspects of RAGE-mediated alteration of lipid synthesis and metabolism.

Our observation that the PI3K-Akt signaling pathway was significantly enriched in DEGs at multiple time points in RAGE KO and WT animals exposed to SHS or RA was somewhat expected. This agrees with prior work showing that the effect of RAGE on the PI3K-Akt pathway can induce autophagy and apoptosis [35,36,37]. We also found the NF-kappa B signaling pathway to be significantly affected in the WT + SHS vs. WT + RA at six months and the WT + RA animals at six months vs. the WT + RA animals at three months in our system, which also agrees with previous general findings [38,39,40]. Of particular interest is the discovery that the MAPK signaling pathway was a prominent axis clarified by KEGG pathway analysis (Figure 3C). We have previously identified increased Ras activation, a molecule GTPase that perpetuates RAGE signing into the cell following interaction with ligands [41]. Furthermore, this previous work revealed RAGE-mediated activation of NF-kB and a role for RAGE in the secretion of numerous pro-inflammatory cytokines including IL-13, Eotaxin, MIP-1γ, IFN-γ, Lymphotaxin, MCP-1, MCSF, MIG, TECK, TNF-α, sTNR-R1, and sTNF-RII following SHS exposure [41]. Our confirmation that MAPK signaling is a central pulmonary response to SHS foreshadows at least 113 additional gene targets that likely orchestrate responses to exposure.

We believe it is important to point out at least a subset of the possible limitations of the current study. First, the animal model that was characterized in this study does suggest multiple potential roles of RAGE in SHS exposure; however, future work will be needed to determine whether these changes are replicated in other model systems and in humans. Second, although RNAseq is highly quantitative and some of our results concur with prior work, future validation experiments will serve to confirm our findings. Lastly, our study was focused on the role of RAGE in the context of SHS exposure, with additional experiments needed to determine its role in other respiratory conditions. These data collectively identify several opportunities to further dissect RAGE signaling in the context of SHS exposure and foreshadow possible therapeutic modalities.

## 4. Materials and Methods

### 4.1. Mice and SHS Treatments

Female mice in a C57Bl/6 background were maintained in a pathogen-free environment under a 12 h light/dark cycle with unrestricted access to food and water. Adolescent mice, aged 40 days (PN40), were subjected to either secondhand smoke (SHS) or ambient room air (RA) over a 6-month span (about 24 weeks). Both wild-type (WT) and RAGE knockout (RKO) mice, (*n* = 4 per group) experienced SHS exposure using a nose-only inhalation system (InExpose System, Scireq, Montreal, QC, Canada), following protocols outlined in prior research [41,42]. Exposure sessions were conducted five days per week, based on preliminary experiments and existing studies that determined the necessary duration to induce physiological changes. Mice in the RA control groups were also restrained and exposed to room air under similar conditions, with no fatalities reported during the study among animals in any group. At the end of the 6 months, mice were euthanized; while the left lung was dedicated to obtaining bronchoalveolar lavage fluid (BALF) not described in the current investigation, the non-lavaged right lungs were excised and snap-frozen in liquid nitrogen for subsequent analysis of total RNA. The study protocols were approved by Brigham Young University’s Institutional Animal Care and Use Committee (IACUC), conforming to all relevant guidelines, under protocol number 21-0203, with approval valid from 17 March 2021 to 16 March 2024.

### 4.2. RNA Extraction, Library Preparation, and Sequencing

Total lung RNA was isolated using the Direct-zol RNA MiniPrep kit w/TriReagent (Zymo Research, Irvine, CA, USA, Cat No. R2053-A). Briefly, resected lung tissue was homogenized at high speed in tri reagent and then centrifuged. Supernatants were collected and then mixed with equal parts of 100% ethanol before being transferred to a zymo-spin IICR column. DNase I treatment was performed as recommended in the manufacturer’s protocol, and all other column filtration washes were performed according to instructions included in the kit. After the washes, RNA was eluted with 25 μL of DNase/RNase-Free Water, and then stored at −80 °C until library preparation. The mRNA from the samples was purified in preparation for cDNA synthesis using poly-dT oligo-attached magnetic beads. First-strand cDNA was subsequently synthesized using random hexamer primers, followed by second-strand cDNA synthesis, end repair, A-tailing, Illumina adapter ligation, size selection, amplification, and purification. The sequencing library was then quantified using a Qubit instrument (Fisher Scientific, Waltham, MA, USA) and real-time PCR, and a Bioanalyzer (Fisher Scientific) was used to characterize size distribution. Paired-end Illumina sequencing was then performed on the normalized and pooled barcoded quantified libraries using an Illumina NovaSeq instrument (Novogene, Sacramento, CA, USA).

### 4.3. Read Mapping, Quantification, and Differential Expression

The paired-end sequencing reads were trimmed for quality and adapters with commercial software. Read mapping was performed using Hisat2 (version 2.0.5) [43] using the indexed mus musculus reference genome and annotation files from Ensembl (version 107) [44]. The featureCounts software (version 2.0.6) was then used to count the number of reads that mapped to the reference genome, with over 90% of reads uniquely mapped [45]. The read counts were then normalized to fragments per kilobase of transcript sequence per million (FPKM) method. Differential expression analysis was performed using the DESeq2 algorithm (R package version 1.20) with genes having a false-discovery rate-adjusted *p*-value < 0.05 categorized as statistically significant [46].

### 4.4. Functional Enrichment Analyses

The clusterProfiler method and Gene Set Enrichment Algorithm (GSEA) were used to perform the KEGG and Reactome intracellular signaling pathway enrichment as well as the Gene Ontology (GO) enrichment analysis [47,48,49,50,51], with adjusted *p*-values < 0.05 categorized as statistically significant. Similar enrichments for diseases (using the Disease Ontology) and gene-disease associations (using DisGeNET data) were also performed.

## Figures and Tables

**Table 1:** Metadata for Analyzed Samples.

Genotype	Exposure Type	Duration of Exposure	Abbreviation	Sample Size
Wild-type (WT)	Secondhand smoke (SHS)	3 Months	WT_SHS_3m	*n* = 4
		6 Months	WT_SHS_6m	*n* = 4
	Room air (RA)	3 Months	WT_RA_3m	*n* = 4
		6 Months	WT_RA_6m	*n* = 4
RAGE Knockout (RKO)	Secondhand smoke (SHS)	3 Months	RKO_SHS_3m	*n* = 4
		6 Months	RKO_SHS_6m	*n* = 4
	Room air (RA)	3 Months	RKO_RA_3m	*n* = 4
		6 Months	RKO_RA_6m	*n* = 4

## Data Availability

All data are presented within the article. Data and other materials are available from the corresponding author on reasonable request.

## Supplementary Materials

The supporting information can be downloaded at https://www.mdpi.com/article/10.3390/ijms25094940/s1.
